# Ultrasonic optic nerve sheath diameter can be used as a diagnostic measure after accidental dural puncture during cesarean section: a case report

**DOI:** 10.1186/s12871-024-02418-8

**Published:** 2024-01-22

**Authors:** Pei Wang, Xia Zhou, Fang Sheng, Xiaolong Wang, Caifeng Shi, Wei Feng

**Affiliations:** 1 Department of Anesthesiology, The Affiliated Hospital of Qingdao University, Qingdao, 266000 China; 2 Department of Emergency surgery, The Affiliated Hospital of Qingdao University, Qingdao, 266000 China

**Keywords:** Cerebral venous thrombosis, Postdural puncture headache, Optic nerve sheath diameter, Cesarean section

## Abstract

**Background:**

Parturients are prone to postdural puncture headache (PDPH) after epidural puncture. Cerebral venous sinus thrombosis (CVST) is a fatal complication of PDPH. The main symptom of both is headache, however, the mechanism is not similar. For persistent PDPH, early differential diagnosis from CVST is essential. Optic nerve sheath diameter (ONSD) measurements can be used to identify changes in intracranial pressure as an auxiliary tool to distinguish the cause of headache.

**Case presentation:**

The dura of a 32-year-old woman undergoing cesarean section was accidentally penetrated while administering epidural anesthesia, and the patient developed PDPH the subsequent day. The patient refused epidural blood patch (EBP) treatment and was discharged after conservative treatment. Fourteen days post-discharge, she was readmitted for a seizure. Magnetic resonance imaging (MRI) and Magnetic resonance angiography (MRA) indicated low cranial pressure syndrome and superior sagittal sinus thrombosis with acute infarction. The next morning, the EBP was performed with 15 ml autologous blood. Subsequently, the headache symptoms decreased during the day and worsened at night. ONSD measurement suggested dilation of the optic nerve sheath, and subsequently, the patient showed intracranial hypertension with papilledema. After dehydration and anticoagulant treatment, the patient’s symptoms were relieved and she was discharged from the hospital 49 days later.

**Conclusions:**

Headache is the main symptom of PDPH and cerebral venous thrombosis, which are difficult to distinguish. ONSD measurement may help to estimate the intracranial pressure, and early measurement may be helpful for women with PDPH to avoid serious complications, such as CVST.

## Background

Postdural puncture headache (PDPH) is a frequently encountered complication of intraspinal anesthesia, particularly among obstetric patients, owing to various factors, such as sex, age, and pregnancy status. The incidence of dural rupture resulting from intraspinal anesthesia in obstetrics ranges from 0–2.6% [1]. The risk of PDPH once accidental dural puncture (ADP) occurs is approximately 50–88% [2]. PDPH mainly occurs due to a decrease in the intracranial pressure (ICP). Hence, currently, once ADP occurs, conservative treatment measures, such as bed rest, fluid supplementation, the utilization of non-steroidal drugs and opioids, antiemetic drugs, the administration of caffeine, and epidural blood transfusions, all of which aim to dilate cerebral vascular contractions and augment cerebrospinal fluid (CSF) production should be considered [2–4].

However, severe PDPH may cause various complications, such as chronic headaches, reversible cerebrovascular systolic syndrome, subdural hemorrhage, intracranial hemorrhage, and cerebral venous sinus thrombosis (CVST) [1]. CVST has a high mortality after PDPH, and the main initial symptom is also headache; however, the headache is due to increased ICP [5]. For persistent PDPH, CVST should be considered as a differential diagnosis, however, simple differential tools to aid diagnosis are lacking.

Herein, we present a case of headache after ADP during cesarean section, who subsequently developed CVST. Studies have shown that patients with PDPH exhibit a reduced optic nerve sheath diameter [6]. Conversely, during the treatment, subsequent to the complications associated with CVST, our patient displayed a dilatation of the optic nerve sheath. Consequently, we explored the potential of utilizing intracranial optic nerve sheath measurement as a diagnostic criterion to effectively prevent further complications in cases of ADP.

## Case presentation

**Fig. 1:**
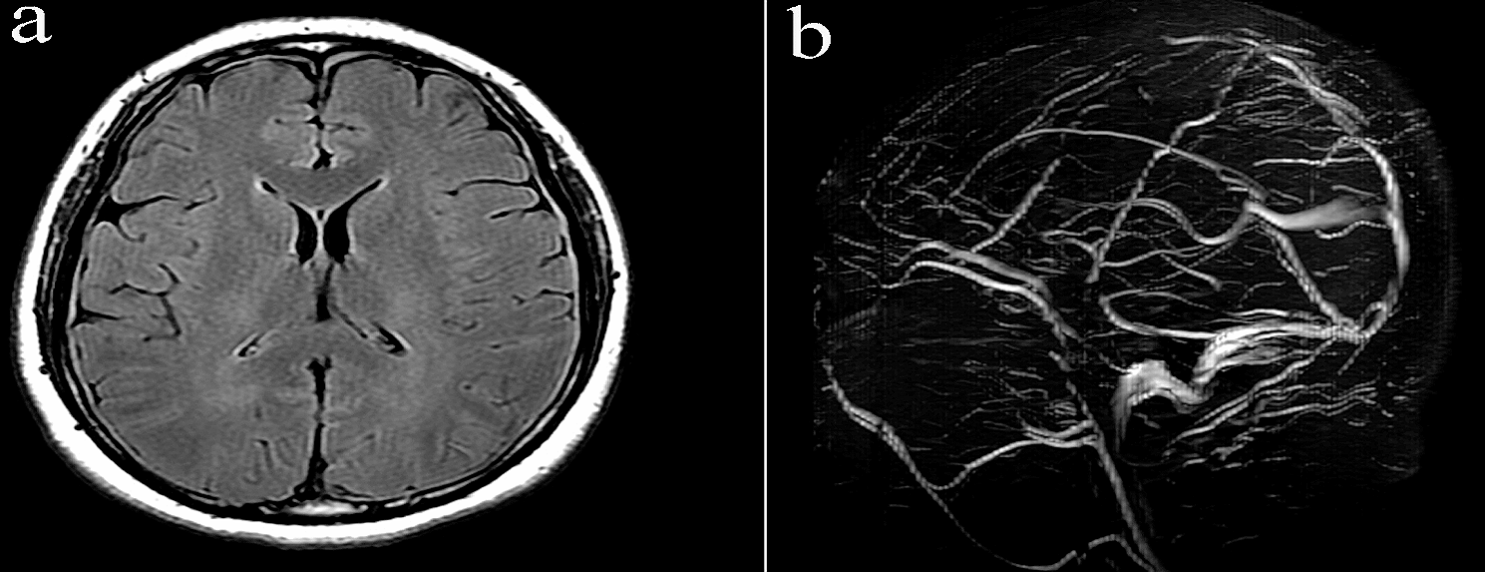
Craniocerebral MRI scan showed narrowing or disappearance of the sulci, thickening and enhancement of the dura (**a**). The brain MRV showed that the swollen left parietal cortex, venous thrombosis (part of the superior sagittal sinus and local draining vein), possible venous infarction of the left parietal lobe, and the indistinct superior sagittal sinus (**b**). MRI: Magnetic Resonance Imaging; MRV: Magnetic Resonance Venography

**Fig. 2:**
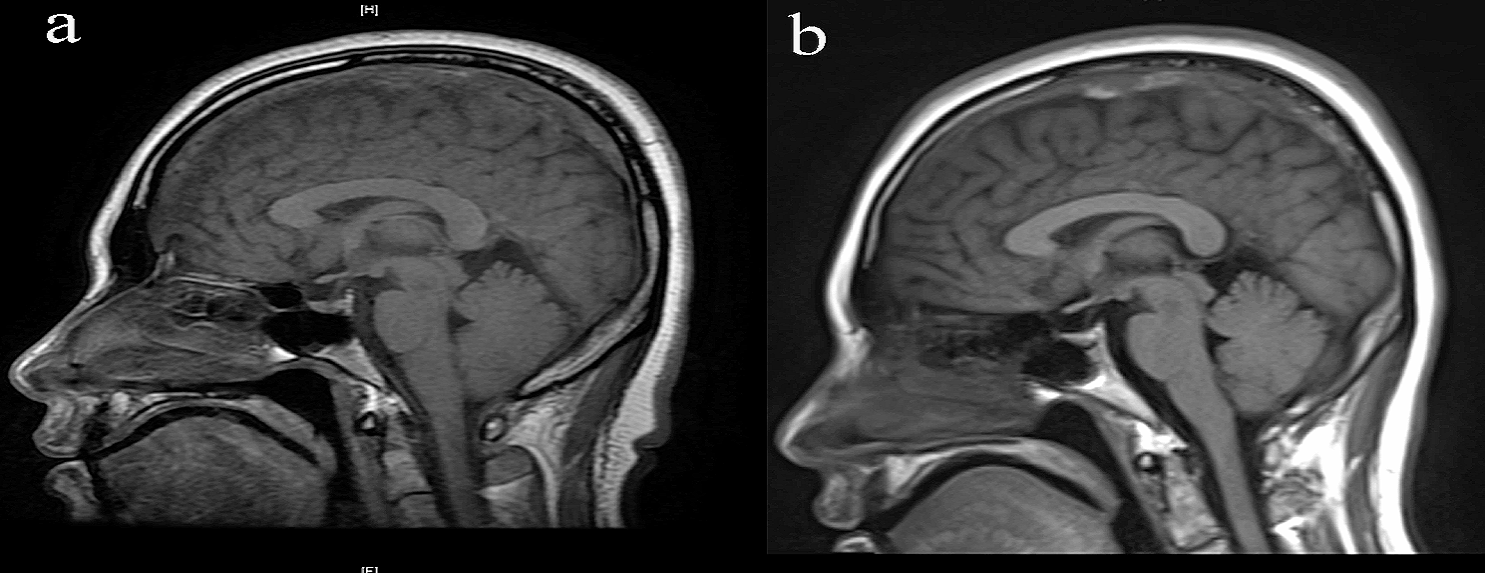
Compared with Figure a, the unclear portion of the superior sagittal sinus had no significant change, but the left parietal cortex swelling was decreased. **a**: the 1st day after admission (before EBP); **b**: the 3rd day after admission (after EBP). EBP: epidural blood patch

**Fig. 3:**
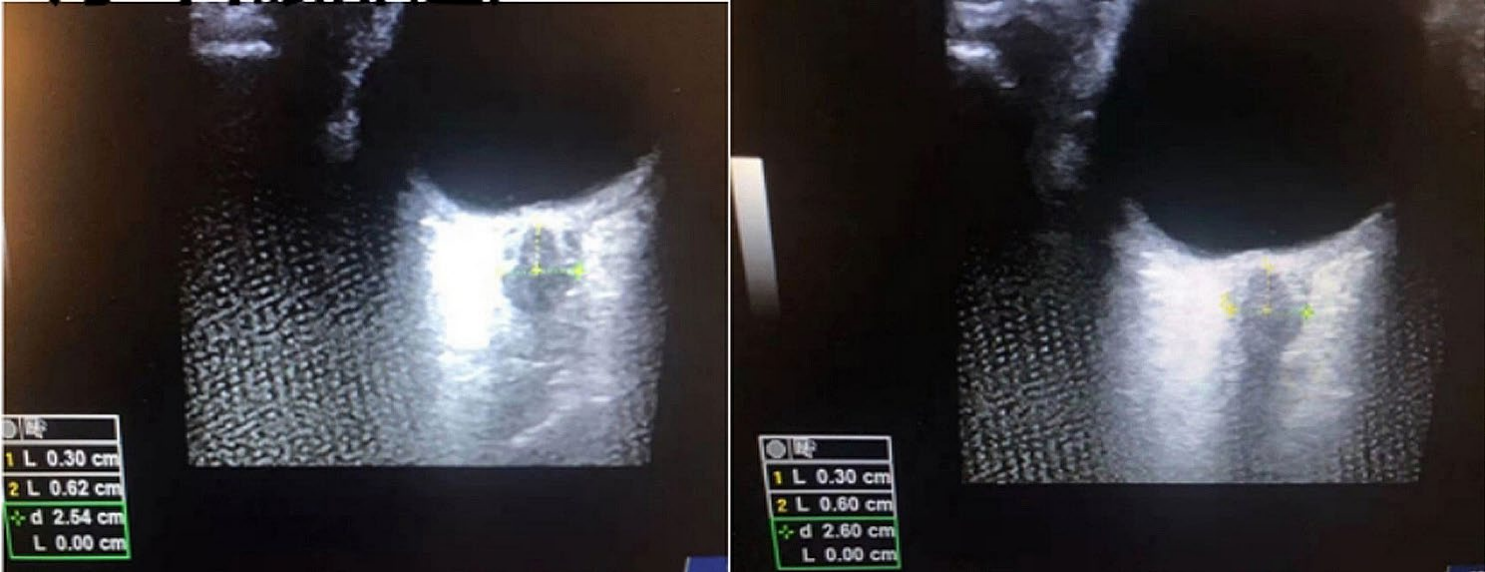
The ONSD (yellow line) was measured 3 mm behind the eyeball and perpendicular to the linear axis of the optic nerve. The ONSD was 6.0 mm on the left and 6.2 mm on the right side. ONSD: optic nerve sheath diameter

A 32-year-old female (height 175 cm, weight 97 kg) presented to the emergency room of our hospital with a seizure. The patient reported that she was a hepatitis B virus carrier, and had undergone a cesarean section with epidural anesthesia 2 weeks ago. The epidural puncture was conducted using an 18-gauge needle in the lumbar space between the 2nd and 3rd vertebrae. Unfortunately, the initial puncture was unsuccessful, and the subsequent puncture accidentally breached the dural membrane, resulting in the leakage of cerebrospinal fluid (CSF). The next day, the patient experienced postdural puncture headache (PDPH), which worsened on sitting but improved on lying down. Despite medical recommendations, the patient and their family declined epidural blood patching (EBP) as a treatment option. Hence, after 9 days of conservative treatment involving bed rest and fluid rehydration, the headache subsided, and the patient was discharged. The first day of readmission, a neurological examination revealed no notable abnormalities. Computed tomography revealed the presence of edema in the left parietal cortex, potentially indicating intracranial hypotension syndrome (IHS). Magnetic resonance imaging (MRI) and magnetic resonance angiography (MRA) confirmed the CVST along with left parietal vein infarction (Fig. 1). Laboratory tests indicated an elevated D-dimer level of 1,700 ng/ml (normal range: 0–500 ng/ml), while the prothrombin time (INR) (0.92) and activated partial thromboplastin time (30.10 s) were within normal limit. No other notable laboratory findings were present. The imaging findings presence of a syndrome characterized by decreased cranial pressure. Consequently, on the second day of hospitalization, a local anesthesia was administered to extract 15 ml of autologous blood for conducting epidural blood patch (EBP) therapy. Following EBP therapy, the patient reported a decrease in daytime headaches but an exacerbation of symptoms during nighttime. Three days after admission, MRI revealed improved post-treatment cranial pressure (Fig. 2). Nevertheless, the patient’s headache persisted despite anticoagulant therapy. Four days after admission, the left and right optic nerve sheath diameters (ONSDs) were 6.0 and 6.2 mm, respectively (Fig. 3).

**Fig. 4:**
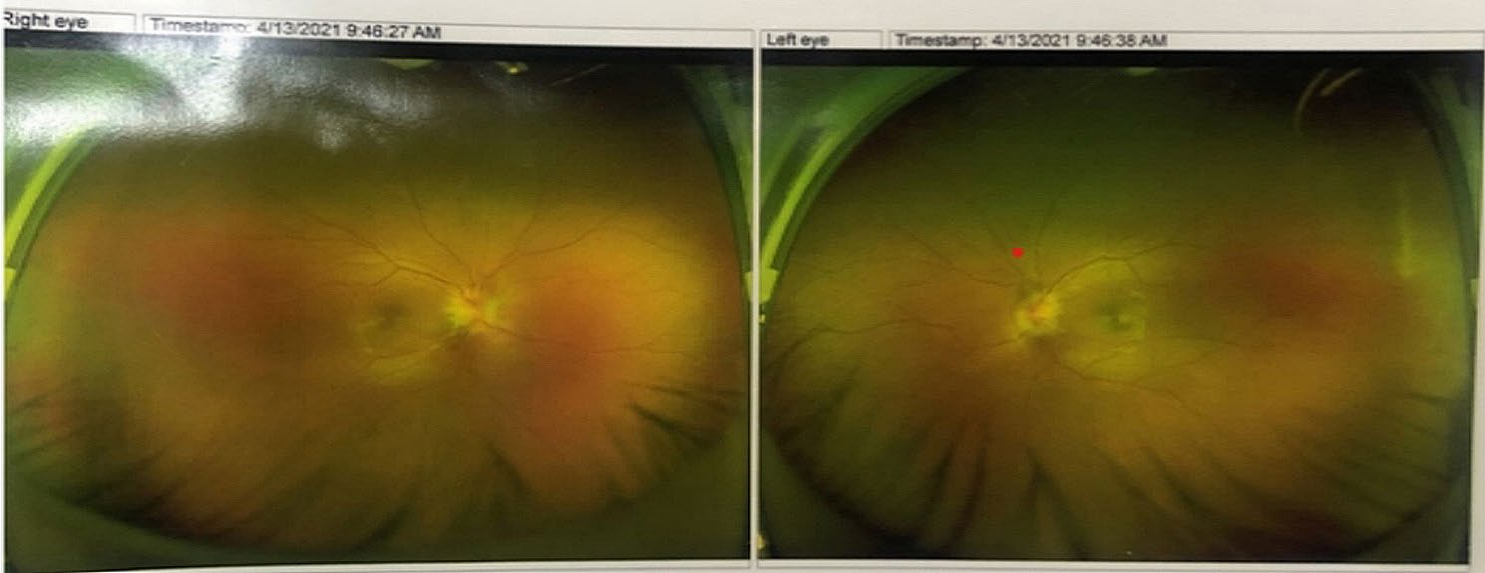
Fundus examination showed papilledema and fundus hemorrhage

Thirteen days after admission, the patient exhibited additional symptoms of blurred vision and a circumscribed dark shadow in the right eye. A fundus examination revealed the presence of papilledema and fundus hemorrhage (Fig. 4). The possibility of intracranial hypertension could not be ruled out. Consequently, the intensity of dehydration treatment and the anticoagulation regimen were adjusted, following which, the patient experienced improvement in visual papilledema, resolution of the fundus bleeding, and restored vision. Subsequently, the patient was discharged after a hospital stay of 49 days.

## Discussion

This case report presents a case of ADP that occurred during cesarean section, which developed into PDPH and subsequently to CVST. Similar cases have been documented in previously [7, 8]. CVST is a rare and fatal complications of the postpartum period, hence, early diagnosis and proper differential diagnosis are crucial.

The predisposing factors for CVST include hypercoagulable states, notably pregnancy, and the prenatal period [9]. Moreover, dural puncture is also linked to the occurrence of CVST. A dural puncture can lead to decreased CSF pressure, potentially causing displacement that impacts cerebral blood vessels and sinuses, thereby causing venous wall deformation or subsequent thrombus formation [7]. CVST symptoms includes headache, focal neurological impairment, seizures, dural arteriovenous fistula, papilledema, intracranial hemorrhage, blurred vision, mental change, diplopia, disturbance of consciousness, and coma [10]. Headache is the predominant clinical manifestation, accounting for 79.0 – 85.4% of cases, and its similarity to PDPH in the early stages often leads to misdiagnosis [7].

For CVST diagnosis, first, a change in headache characteristics accompanied by the absence of postural elements and the presence of focal neurological signs serve as important clinical features for distinguishing CVST-related headaches from PDPH [11]. Second, the diagnostic accuracy of elevated D-dimer levels for the diagnosis of CVST was found to be 94.1% and 97.5% for sensitivity and specificity. Third, even though MRI is the preferred imaging modality, MRA remains the gold standard for diagnosing this syndrome [10, 11]. 

The main symptoms of PDPH and CVST are headaches, however, PDPH headache is caused due to decreased ICP caused by the loss of CSF, and CVST headache is the due to increased ICP. Changes in the volume of CSF may cause changes in ONSD, which are sheathing structures wrapped in the meninges to which CSF can enter freely. Increased or decreased subarachnoid fluid can lead to dilation or narrowing of the ONSD, respectively [12]. 

Research has demonstrated that ultrasonographic ONSD predicts elevated ICP in patients who are unsuitable for invasive ICP measurement or are critically ill [12–14]. One study found a strong positive correlation (R^2^ = 0.80) between ICP and ONSD. Using a cutoff of ≥ 5.0 mm ONSD, the sensitivity and specificity for detecting elevated ICP were 94% and 98% and an area under the curve (AUC) of 0.99 (95% CI 0.97–1.00) [15]. 

Moreover, ONSD has shown some predictive value for PDPH, with the ONSD measurement at T24 being the most accurate predictor (AUC, 0.9787; 95% CI, 0.9578–0.9996), which has a sensitivity and specificity of 92% and 94%, respectively, with a cutoff value of 0.40 cm [6]. Ahmet Besir et al. showed that in patients with headache induced by low ICP, headache severity was negatively correlated with ONSD, and the higher the headache severity, the greater the decrease in ONSD [16]. 

In the present report, after the patient developed PDPH due to an ADP, she was treated conservatively and discharged from the hospital after 9 days with headache relief. Unfortunately, ONSD measurements were not performed during this period. Four days after the patient was readmitted to the hospital with a headache, ONSD measurements showed optic nerve sheath dilation, and nine days later, the patient had elevated ICP symptoms, blurred vision, and papilledema. Although the patient was discharged with improvement after follow-up treatment, our experience of this case suggests that ONSD measurement can be used as a prediction tool for PDPH. If a patient has a persistent headache with no relief, dynamic ONSD measurement can be performed. If the ONSD continues to be < 4 mm, it indicates that the effect of conservative treatment is poor. Blood patches or other measures can be carried out in the meantime. If the patient has an ONSD of > 5 mm, an imaging examination should be conducted timely to avoid CVST and other similar serious complications.

Angiography remains the gold standard for the diagnosis of CVST. However, ONSD has some advantages compared to other imaging examinations. First, the measurement is easy to obtain, especially for women with limited postpartum check-ups, since a bedside ultrasound can be completed, and the cost is lower. ONSD costs 30 ￥each test, MRI costs 300￥each test, and MRA costs 460￥each test in our institution. If the patient has a PDPH, ONSD may be the first choice, and if the headache persists, MRI or MRA should be considered.

However, there are additional limitations associated with the assessment of ONSD as a substitute metric for ICP. These limitations encompass the considerable variability in ONSD truncation values, as well as the impact of factors such as age, sex, and body mass index. More prospective studies are needed to determine this. However, ONSD can be considered as a valuable qualitative approach for assessing persistent headaches after ADP to guide clinical treatment.

## Conclusion

After ADP, if the puerpera has a persistent headache with no relief, by monitoring the dynamic changes of ONSD, early diagnosis and treatment can be carried out to avoid serious complications, such as CVST.

## Data Availability

No datasets were generated or analysed during the current study.

## Abbreviations

PDPH: Postdural puncture headache
CVST: Cerebral venous sinus thrombosis
ONSD: Optic nerve sheath diameter
EBP: Epidural blood patch
ADP: Accidental dural puncture
HIS: Intracranial hypotension syndrome
MRI: Magnetic resonance imaging
MRA: Magnetic resonance angiography
